# Adiposity and the isotemporal substitution of physical activity, sedentary time and sleep among school-aged children: a compositional data analysis approach

**DOI:** 10.1186/s12889-018-5207-1

**Published:** 2018-03-02

**Authors:** Dorothea Dumuid, Tyman E. Stanford, Željko Pedišić, Carol Maher, Lucy K. Lewis, Josep-Antoni Martín-Fernández, Peter T. Katzmarzyk, Jean-Philippe Chaput, Mikael Fogelholm, Martyn Standage, Mark S. Tremblay, Timothy Olds

**Affiliations:** 1 Alliance for Research in Exercise, Nutrition and Activity (ARENA), School of Health Sciences, University of South Australia, GPO Box 2471, Adelaide, SA 5001 Australia; 2 School of Mathematical Sciences, The University of Adelaide, Adelaide, Australia; 3 Institute of Sport, Exercise and Active Living, Victoria University, Melbourne, Australia; 4 School of Health Sciences, Flinders University and School of Health Sciences, University of South Australia, Adelaide, Australia; 5 Department of Computer Science, Applied Mathematics and Statistics, University of Girona, Girona, Spain; 6 Pennington Biomedical Research Center, Baton Rouge, USA; 7 Healthy Active Living and Obesity Research, Children’s Hospital of Eastern Ontario Research Institute, Ottawa, Canada; 8 Department of Food and Environmental Sciences, University of Helsinki, Helsinki, Finland; 9 Department for Health, University of Bath, Bath, England; 10 LBT Innovations, Adelaide, Australia

**Keywords:** Physical activity, sedentary behaviour, sleep, isotemporal substitution, adiposity

## Abstract

**Background:**

Daily activity data are by nature compositional data. Accordingly, they occupy a specific geometry with unique properties that is different to standard Euclidean geometry. This study aimed to estimate the difference in adiposity associated with isotemporal reallocation between daily activity behaviours, and to compare the findings from compositional isotemporal subsitution to those obtained from traditional isotemporal substitution.

**Methods:**

We estimated the differences in adiposity (body fat%) associated with reallocating fixed durations of time (isotemporal substitution) between accelerometer-measured daily activity behaviours (sleep, sedentary time and light and moderate-to-vigorous physical activity (MVPA)) among 1728 children aged 9–11 years from Australia, Canada, Finland and the UK (International Study of Childhood Obesity, Lifestyle and the Environment, 2011–2013). We generated estimates from compositional isotemporal substitution models and traditional non-compositional isotemporal substitution models.

**Results:**

Both compositional and traditional models estimated a positive (unfavourable) difference in body fat% when time was reallocated from MVPA to any other behaviour. Unlike traditional models, compositional models found the differences in estimated adiposity (1) were not necessarily symmetrical when an activity was being displaced, or displacing another (2) were not linearly related to the durations of time reallocated, and (3) varied depending on the starting composition.

**Conclusion:**

The compositional isotemporal model caters for the constrained and therefore relative nature of activity behaviour data and enables all daily behaviours to be included in a single statistical model. The traditional model treats data as real variables, thus the constrained nature of time is not accounted for, nor reflected in the findings. Findings from compositional isotemporal substitution support the importance of MVPA to children’s health, and suggest that while interventions to increase MVPA may be of benefit, attention should be directed towards strategies to avoid decline in MVPA levels, particularly among already inactive children. Future applications of the compositional model can extend from pair-wise reallocations to other configurations of time-reallocation, for example, increasing MVPA at the expense of multiple other behaviours.

**Electronic supplementary material:**

The online version of this article (10.1186/s12889-018-5207-1) contains supplementary material, which is available to authorized users.

## Background

Higher levels of moderate-to-vigorous physical activity (MVPA) and sleep duration in children have been associated with lower adiposity (e.g., body fat%); conversely, greater sedentary time has been associated with higher adiposity [1–3]. More recently, studies have examined the associations between adiposity and the reallocation of a fixed duration of one activity behaviour for another, using the isotemporal substitution methodology proposed by Mekary et al. [4]. Conceptually, the traditional isotemporal substitution paradigm takes the finite nature of daily activity behaviours into account. However, the suitability of this isotemporal model for constrained data has been questioned [5, 6].

The traditional isotemporal substitution method is based on regression modeling. All activity behaviours (min/day) except one are used as explanatory variables in the model, and a term for the total number of measured minutes, i.e. the sum of all activity behaviour variables, is also included. The non-standardised regression coefficient corresponding to each of the activity behaviours included as explanatory variables in the model is considered to be the estimated change in the outcome variable when one minute of that activity behaviour replaces the activity behaviour excluded from the regression model. A more detailed explanation of the traditional isotemporal substitution model can be found elsewhere [4, 7].

An alternative approach for analysing daily activity behaviours — compositional data analysis — was recently suggested by Pedišić [6] and later pioneered in empirical studies by Chastin et al. [8] and Carson et al. [9]. Compositional data analysis conceptualises individuals’ daily activity data as *compositions*, made up of mutually exclusive and exhaustive *parts* (times spent in sleep, sedentary behaviour, light physical activity (LPA) and MVPA), which are constrained by a daily constant sum of 1440 min. Accordingly, these data exist in a constrained space (*the simplex)* governed by a specific geometry (*Aitchison geometry*) [10]. Operations in the simplex differ to those defined for real space, i.e., Euclidean geometry (e.g., the Euclidean sum and product are operationalised as perturbation and powering in the simplex) [10]. Therefore traditional statistical techniques using Euclidean operations (such as multiple linear regression) are incongruent with the Aitchison geometry of the simplex [11]. Compositions can, however be translated to real space through the application of isometric log ratio *(ilr*) coordinate systems [11]. Once compositions are represented as *ilr* coordinates*,* they are governed by Euclidean geometry, and traditional analytical techniques such as linear regression can be used [11].

The *ilr* linear multiple regression model can be used to estimate or predict an outcome of any given composition that has been expressed as *ilr* coordinates [12]. By systematically changing the predictive composition (as *ilr* coordinates) to simulate isotemporal reallocation between activity behaviour-pairs, a series of estimated outcomes can be generated. Estimated adiposity can be calculated for (1) a baseline composition (e.g., the mean composition), and (2) for a new composition, where a set duration of time has been reallocated from one activity behaviour to another, keeping the remaining behaviours constant at their baseline values. The difference in estimated adiposity can then be calculated for the two compositions. The compositional isotemporal substitution technique is not limited to creating estimates only for the substitution of one behaviour (e.g., sedentary time) for one other behaviour (e.g., MVPA), but the behaviour of interest could be substituted by two or more other behaviours (e.g., MVPA and sleep). In fact, the difference in adiposity estimated for any reallocation between daily activity behaviours (eg., sleep, sedentary time, LPA and MVPA) can be assessed. This approach was developed by Dumuid and colleagues [13] and first applied in Fairclough et al [14] on a sample of 169 grade five children in the United Kingdom. It is unknown if and how the results from compositional and traditional isotemporal substitution approaches differ.

This study is the first to estimate the difference in adiposity associated with reallocations of time between daily activity behaviours among children using compositional and traditional isotemporal substitution methods, and to compare the findings obtained from the two approaches.

## Methods

### Participants

Participants were from four study sites (Australia, Canada, United Kingdom and Finland) included in the larger cross-sectional 12-nation International Study of Childhood Obesity, Lifestyle and the Environment (ISCOLE) [15]. The four sites were chosen based on previous findings suggesting relative homogeneity of lifestyle behaviours among children at these sites [16]. Site investigators aimed to recruit a sex-balanced sample of 500 children from each site, based on *a priori* power calculations [15]. Children aged 9–11 years (*n* = 2156) were recruited from schools in urban and suburban settings, and stratified by socioeconomic status [15]. Data were collected between September 2011 and December 2013. Children were excluded if they had incomplete data for any of the following parameters; activity behaviours (*n* = 319), as well as body fat% (*n* = 28) and covariates (highest education level of either parent, number of parents and siblings in the home; *n* = 81), resulting in a final sample of 1728 children.

### Ethics

The Pennington Biomedical Research Center in Baton Rouge, Louisiana coordinated ISCOLE and received ethical approval for the overall study protocol from their Institutional Review Board. The four sites also received approval from local ethics committees and school boards. Parents provided signed informed consent and child assent was attained prior to inclusion in the study. ISCOLE is registered on ClinicalTrials.gov, Identifier: NCT01722500.

### Daily activity behaviours

Activity behaviours were measured during the school year over 7 days, by 24-h accelerometry. Actigraph GT3X+ Accelerometers (ActiGraph LLC, Pensacola, FL) were worn on the right hip, with instruction to remove the device only for water-based activities. Wear time compliance in ISCOLE was high (daily average = 22.8 h). Participants were required to have at least 4 days of valid accelerometry data (with ≥10 h of waking wear/day, including at least one weekend day [17]). Data were downloaded in 1-s epochs using the low-frequency extension filter (ActiLife software version 5.6). A fully automated algorithm was used to estimate nocturnal sleep duration (from data aggregated to 60-s epochs) [18, 19]. Waking behaviours were determined (from data aggregated to 15-s epochs), once nocturnal sleep time and awake non-wear time (any sequence of ≥20 consecutive minutes of 0 activity counts) were removed. Evenson’s cut-points were used to define sedentary time (≤25 counts per 15 s), LPA (26–573 counts per 15 s), and MVPA (≥574 counts per 15 s) [20]. These cut-points have been shown to provide acceptable classification accuracy for 5–15 year old children [21]. The daily average for each part of the composition (sleep, sedentary time, LPA and MVPA) was weighted for week:weekend days at 5:2.

### Adiposity

Body fat% was used to indicate children’s overall adiposity. Body fat% was measured during school visits using a portable Tanita SC-240 bioelectrical impedance scale (TANITA Corporation, Tokyo, Japan) [22]. Two measurements were taken, and the average was used in analysis (if measurements were >2.0% apart, a third measurement was taken, and the closest two were averaged for analysis). Bioelectrical impedance analysis is a valid (r = 0.69–0.79) and reliable (CV_intra_ = 3%, SEM_intra_ = −0.91 to 1.61 body fat%) estimate of body fat for school-age children compared to gold standards of underwater weighing and dual-energy X-ray absorptiometry (DXA) [23, 24]. The Tanita SC-240 has acceptable accuracy for estimating body fat% in this age group when assessed against DXA [22]. Body mass index (BMI) z-score, used to classify children into weight-status sub-groups, was calculated from measured height (Seca 213 portable stadiometer, Hamburg, Germany) and weight (TANITA Corporation, Tokyo, Japan [22]), using BMI = weight (kg)/height (m^2^) and age- and sex-specific World Health Organization reference data [25] for standardisation as z-scores.

### Covariates

The highest education level of either parent, and the number of parents and siblings in the home were reported by parents [15]. These sociodemographic factors were selected as covariates due to their potential influence on both activity behaviours and adiposity [26–28].

### Analysis

Analysis was conducted in R (R Development Core Team, Vienna, Austria), using the packages Compositions [29] and robCompositions [30]. The daily behaviour composition was described in terms of centre (geometric mean of each behaviour, adjusted to collectively sum to 1440 min) [8, 31] and dispersion (variation matrix) [8, 10]. Analysis consisted of exploring the relationship between isotemporal substitution of one behaviour for another behaviour and children’s body fat%, using (i) traditional isotemporal substitution [4] and (ii) compositional isotemporal substitution. All analyses were stratified by sex.

Traditional isotemporal substitution analysis followed the procedure outlined by Mekary et al. [4]. Four linear regression models were created with body fat% as the dependent variable. The explanatory variables of the first model included each daily behaviour (sedentary time, LPA, MVPA) except one (sleep). Total time was also included; with the effect of constraining the linear model so that the regression coefficients represented the estimated change in adiposity associated with the substitution of one unit of sleep (i.e., 30 min) for one unit of the corresponding remaining behaviours. To capture all isotemporal eventualities, the remaining three linear models each omitted a different behaviour, while adjusting for all remaining behaviours, total wear time and covariates.

Compositional isotemporal substitution used multiple linear regression models with the activity behaviour composition (expressed as *ilr* coordinates) and sociodemographic covariates as explanatory variables. A comprehensive explanation of the compositional analysis and sample R code is included in Additional file 1. The *ilr* model was used to estimate body fat% at a baseline composition (e.g., the mean daily activity behaviour composition), and subsequently at new compositions (e.g., sleep = mean sleep +30 min, sedentary time = mean sedentary −30 min, LPA = mean LPA and MVPA = mean MVPA). The estimated absolute differences in body fat percentage units was calculated using predictions from the model (i.e., estimated difference in body fat% = estimated body fat% at baseline composition – estimated body fat% at new composition). Estimates were calculated for time reallocations of 30 min. Effect sizes (ES) were calculated by dividing the estimated differences by the mean standard deviation of body fat%. Additionally, estimated changes in body fat% across the continuum of reallocations in 5-min increments up to 70 min were graphically illustrated, using MVPA as an example.

This method of compositional isotemporal substitution uses a different approach from the change-matrix procedure outlined in Chastin et al. [8], where pairwise log ratios obtained by applying the inverse *ilr* transformation to the regression coefficients are introduced to facilitate the interpretation of the change in response variable. Each of these pairwise log-ratios is analogous to one of the three *ilr* coordinates of a four-part composition. However, the remaining *ilr* will necessarily contain information regarding the two components involved in the pair-wise log ratio. The method in Chastin et al. [8] is a useful tool, but prediction and inference are more comprehensive when made using the complete set of *ilr* coordinates.

Compositional isotemporal substitution was conducted in sex-stratified cohorts, at the daily behaviour mean composition (geometric mean of each behaviour, adjusted to collectively sum to the daily total of 1440 min) for all children, and repeated at the daily mean composition for (i) normal weight children (−2 ≤ zBMI≤1); (ii) overweight children (1 < zBMI≤2); and (iii) obese children (zBMI>2) [25].

## Results

Included participants were more likely to be girls (*P* = 0.02), have parents of higher education (*P* = 0.04) and fewer siblings (*P* < 0.001) than excluded participants (Additional file 2). They also had different mean activity behaviour compositions (*P* < 0.001), but not body fat% (*P* = 0.27). Daily averages of sleep, sedentary time, LPA and MVPA for boys and girls and variation matrices describing relative dispersion among pair-wise behaviours are included in Additional file 3.

### Traditional isotemporal substitution

The absolute differences in estimated body fat% appeared to be the greatest when time was reallocated between LPA and MVPA (boys) or either LPA or sedentary time and MVPA (girls) (Table 1).

**Table 1:** Traditional and compositional isotemporal substitution: Estimated absolute differences in body fat% associated with 30-min pair-wise reallocations between activity behaviours

BOYS *n* = 760									
Model	Body fat%	Sleep		SED		LPA		MVPA	
	30 min	*∆‘*	95% CI	*∆‘*	95% CI	*∆‘*	95% CI	*∆‘*	95% CI
Traditional	Sleep			−0.29	−0.56, −0.01	−0.56	−0.92, −0.20	1.38	0.78, 2.00
Compositional				−0.20	−0.46, 0.05	−0.53	−0.87, −0.18	2.11	1.37, 2.85
Traditional	SED	0.29	0.01, 0.56			−0.27	−0.58, 0.03	1.67	1.13, 2.22
Compositional		0.20	−0.05, 0.46			−0.33	−0.63, −0.02	2.31	1.62, 2.99
Traditional	LPA	0.56	0.20, 0.92	0.27	−0.03, 0.58			1.95	1.26, 2.63
Compositional		0.49	0.16, 0.83	0.28	−0.01, 0.57			2.60	1.78, 3.42
Traditional	MVPA	−1.38	−2.00, −0.78	−1.67	−2.22, −1.13	−1.95	−2.63, −1.26		
Compositional		−1.33	−1.83, −0.82	−1.54	−1.99, −1.09	−1.86	−2.46, −1.26		
GIRLS *n* = 968									
Model	Body fat%	Sleep		SED		LPA		MVPA	
	30 min	*∆‘*	95% CI	*∆‘*	95% CI	*∆‘*	95% CI	*∆‘*	95% CI
Traditional	Sleep			−0.55	−0.85, −0.25	−0.49	−0.85, −0.12	1.60	0.80, 2.39
Compositional				−0.52	−0.80, −0.24	−0.49	−0.85, −0.12	2.44	1.34, 3.54
Traditional	SED	0.55	0.25, 0.85			0.07	−0.25, 0.38	2.15	1.41, 2.89
Compositional		0.52	0.24, 0.80			0.02	−0.30, 0.34	2.94	1.89, 4.00
Traditional	LPA	0.49	0.12, 0.85	−0.07	−0.38, 0.25			2.08	1.20, 2.97
Compositional		0.47	0.13, 0.82	−0.06	−0.36, 0.24			2.90	1.70, 4.10
Traditional	MVPA	−1.60	−2.39, −0.80	−2.15	−2.89, −1.41	−2.08	−2.97, −1.20		
Compositional		−1.24	−1.88, −0.61	−1.78	−2.36, −1.19	−1.74	−2.50, −0.99		

Abbreviations: *∆****‘*** estimated difference in body fat%, *CI* confidence interval, *SED* sedentary time, *LPA* light physical activity, *MVPA* moderate-to-vigorous physical activity. Analysis adjusted for parental education level and number of parents and siblings. Note: Difference in body fat% is estimated for the reallocation of time from the behaviour in the column to the behaviour in the row, i.e., the first value of −0.29 in Row 1 is the estimated difference in body fat% for the reallocation of 30 min from sedentary time to sleep among boys. Boys’ mean daily activity composition (min/day): Sleep = 550; SED = 510; LPA = 308; MVPA = 72. Mean body fat% for boys: $\overline{x}$ = 18.1. Girls’ mean daily activity composition (min/day): Sleep = 564; SED = 520; LPA = 301; MVPA = 55. Mean body fat% for girls: $\overline{x}$ = 22.3

### Compositional isotemporal substitution

**Fig. 1:**
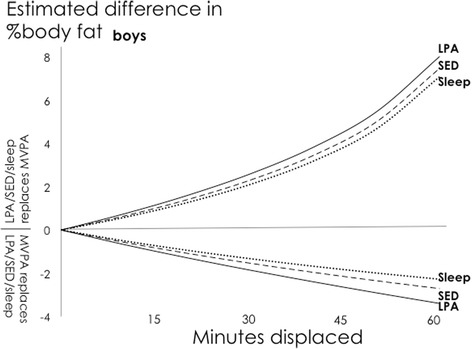
Boys: Estimated absolute difference in body fat% associated with pair-wise reallocations of time between MVPA and other behaviours. Abbreviations: SED, Sedentary Time; LPA, Light Physical Activity; MVPA, Moderate-to-Vigorous Physical Activity. Mean daily behaviour composition for boys (min/day): Sleep = 550, SED = 510, LPA = 308, MVPA = 72

**Fig. 2:**
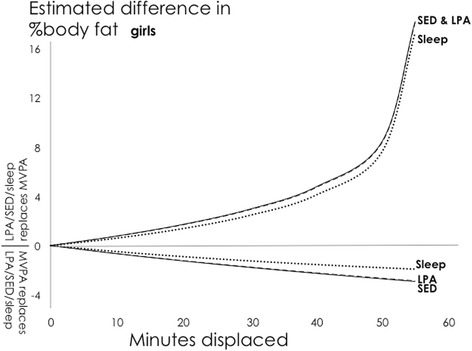
Girls: Estimated absolute difference in body fat% associated with pair-wise reallocations of time between MVPA and other behaviours. Abbreviations: SED, Sedentary Time; LPA, Light Physical Activity; MVPA, Moderate-to-Vigorous Physical Activity. Mean daily behaviour composition for girls (min/day): Sleep = 564, SED = 520, LPA = 301, MVPA = 55

For both sexes, higher estimated body fat% was associated with the reallocation of time from MVPA to other behaviours (Table 1). Notably, the estimated difference in adiposity was not exactly symmetrical, e.g., a reallocation of 30 min from MVPA to sedentary time was associated with a larger estimated absolute difference in body fat% (+2.31 ES = 0.36 for boys; +2.94 ES 0.41 for girls) than when 30 min was reallocated from sedentary to MVPA (−1.54 ES = 0.24 for boys; −1.78 ES = 0.25 for girls). For boys, the reallocation of any given time spent in MVPA to LPA tended to predict the largest positive (unfavourable) difference in body fat% (Fig. 1), whereas for girls, the reallocation of any given time spent in MVPA to either sedentary time or LPA appeared to predict the largest positive difference in body fat% (Fig. 2).

**Fig. 3:**
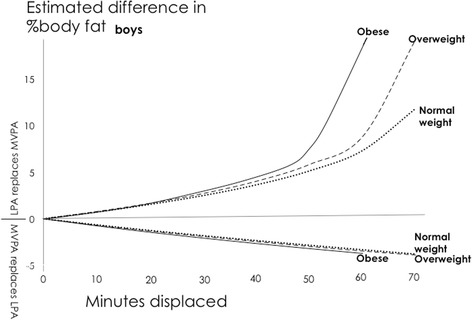
Boys: Estimated absolute difference in body fat% associated with pair-wise reallocations of time between MVPA and LPA around three different daily behaviour mean compositions; normal weight, overweight and obese. Abbreviations: LPA, Light Physical Activity; MVPA, Moderate-to-Vigorous Physical Activity. Mean boys’ daily behaviour composition (min/day): Normal weight: Sleep = 551, SED = 506, LPA = 307, MVPA = 75; Overweight: Sleep = 549, SED = 515, LPA = 307, MVPA = 70; Obese: Sleep = 541, SED = 526, LPA = 313, MVPA = 61

**Fig. 4:**
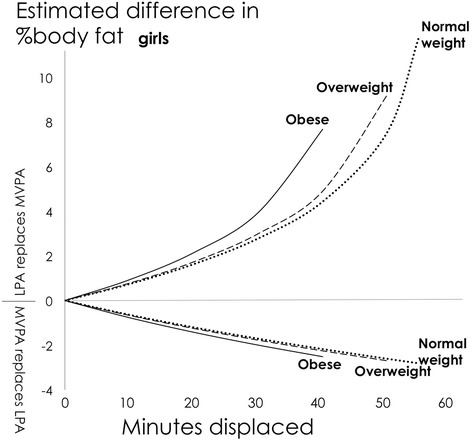
Girls: Estimated absolute difference in body fat% associated with pair-wise reallocations of time between MVPA and LPA around three different daily behaviour mean compositions; normal weight, overweight and obese. Abbreviations: LPA, Light Physical Activity; MVPA, Moderate-to-Vigorous Physical Activity. Mean girls’ daily behaviour composition (min/day): Normal weight: Sleep = 567, SED = 515, LPA = 301, MVPA =57; Overweight: Sleep = 562, SED = 526, LPA = 298, MVPA = 54; Obese: Sleep = 543, SED = 545, LPA = 307, MVPA = 45

Reallocations modeled at the mean daily behaviour composition for different weight status subgroups (Additional file 4) demonstrated larger differences in estimated body fat% for overweight and obese children, compared to those classified as ‘normal weight’, particularly when the reallocated behaviour was MVPA (Figs. 3 and 4).

### Comparison between traditional and compositional isotemporal substitution

**Fig. 5:**
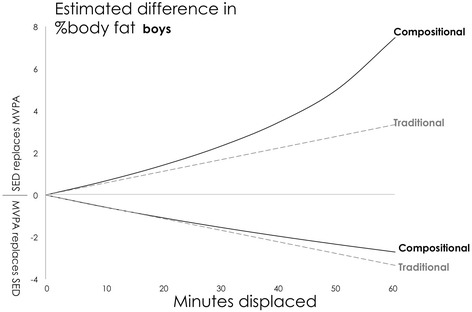
Boys: Estimated absolute difference in body fat% associated with pair-wise reallocations between MVPA and sedentary time as estimated by compositional and traditional isotemporal substitution. Abbreviations: SED, Sedentary Time; MVPA, LPA = Light Physical Activity; Moderate-to-Vigorous Physical Activity. Mean daily behaviour composition for boys (min/day): Sleep = 550, SED = 510, LPA = 308, MVPA = 72

**Fig. 6:**
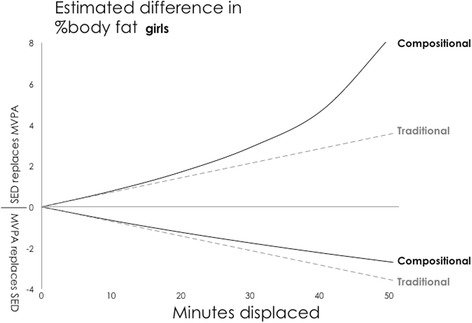
Girls: Estimated absolute difference in body fat% associated with pair-wise reallocations between MVPA and sedentary time as estimated by compositional and traditional isotemporal substitution. Abbreviations: SED, Sedentary Time; MVPA, LPA = Light Physical Activity; Moderate-to-Vigorous Physical Activity. Mean daily behaviour composition for girls (min/day): Sleep = 564, SED = 520, LPA = 301, MVPA = 55

Both statistical techniques found that the largest differences in estimated body fat% were associated with reallocations of time away from MVPA. However, there were some important differences between the findings from the two approaches. The relative difference between the estimates for 30-min reallocations ranged from 0.2% to 121% (median = 18%) (calculated as the absolute difference between estimates from traditional and compositional models divided by a pooled estimate from both models). Larger absolute differences between the traditional and compositional models seem to be associated with higher estimates. Compositional isotemporal substitution findings also differed from traditional isotemporal substitution findings in the following ways: (i) the amount of difference in estimated body fat% when a behaviour was higher or lower was not exactly symmetrical, (Figs. 5 and 6), (ii) the difference in estimated body fat% was not linearly proportional to the amount of time reallocated (Figs. 5 and 6), and (iii) traditional isotemporal models indicated the estimated change in a continuous outcome was associated with same time reallocations irrespective of the starting/reference durations of activity behaviours (in other words, estimated change in body fat% was identical when 30 min of MVPA was reallocated from a starting duration of, for example, either 0 min/day or 150 min/day, whilst in contrast, estimates of change in outcome from compositional models were relative to a starting/reference composition, thus respecting the relative nature of compositional data [Figs. 3 and 4]).

## Discussion

### Main findings

This study estimated the difference in adiposity associated with the isotemporal substitution of daily activity behaviours. For both traditional and compositional models, the greatest differences in body fat% were estimated for the reallocation of time from MVPA to other behaviours, and vice versa. The estimated differences in body fat% for 30-min reallocations with MVPA were relatively modest, with standardised effect sizes ranging between 0.2 and 0.4.

There appeared to be sex differences between the findings. Both models estimated favourable absolute differences in body composition when sedentary behaviour replaced LPA (significant only in the compositional model) among boys, but not among girls. Furthermore, among boys estimated body composition was not most favourable when MVPA replaced sedentary time, whereas it was among girls. This unexpected finding may be an artefact of the cross-sectional study design; there may be a sub-population of lean boys who accumulate large amounts of sedentary time. A number of previous studies have detected clusters of boys with high screen time [32–34]. Interestingly, membership of these clusters was not associated with higher adiposity.

There were some similarities between findings from traditional and compositional models, however there were fundamental differences between the two analytical approaches. The distinguishing feature of the compositional approach is that the inherent compositional nature and corresponding geometry of the data are not violated by the mathematical operations applied [12].

### Differences between traditional and compositional methods

Unlike traditional isotemporal substitution, the compositional method produces asymmetrical estimates. That is, a quantum reallocation of one behaviour for another does not predict the exact inverse effect as the reverse reallocation. In the present study, asymmetry was most notably observed for reallocations with MVPA. Similar asymmetry with reallocation of MVPA was reported for other adiposity indicators by Fairclough et al. [14]. Essentially, adding MVPA appears to yield lower returns, while reducing MVPA seems to be associated with higher expected differences in fatness. In addition, the amount of time reallocated between behaviours was not linearly related to the difference in estimated body fat%. These findings are consistent with dose-response curves observed in experimental studies (e.g. Sattelmair et al. [35]), where marginal increases in exposures regularly predict diminishing benefits [36]. The non-linearity of findings has two implications. Firstly, they suggest that adiposity interventions should encourage children (particularly those with low levels of physical activity) to be more physically active. Secondly, the asymmetry in the estimated dose-response curves suggests that interventions aimed at defending (i.e. maintaining) MVPA may be of great importance, even if MVPA is not increased. However, the cross-sectional nature of the findings and the relatively modest effect sizes should be noted when considering the implications of this study.

### Strengths and limitations

This study has drawn on a large multi-national dataset using standardised measurements, including accelerometer-based measures of activity behaviours and objective measures of body fat% [15]. We used compositional data analysis techniques to quantify the adiposity association of isotemporal substitutions of activity behaviours.

Nonetheless, the study has several limitations. Accelerometers cannot distinguish completely between sitting and standing postures, with both potentially being classified as sedentary time. Whilst the analyses were adjusted for sociodemographic variables, there may be potential residual confounding from other lifestyle, social and environmental factors, such as diet, parental influence and perceived neighbourhood safety that were not included as covariates in our analyses. Furthermore, in ISCOLE, the participants were sampled from urban and suburban centres and were not nationally representative, limiting the generalisability of results [37]. Most importantly, due to the cross-sectional nature of the present isotemporal substitution analyses, findings are more correctly conceptualised as snapshots of adiposity associations with various daily behaviour compositions, than as adiposity associations of various behaviour substitutions within one day. It is therefore not possible to interpret the findings as the effect on adiposity for isotemporal substitutions of time between activity behaviours.

## Conclusions

There were important differences between the two methods. Traditional isotemporal substitution did not account for (i) possible asymmetry of estimated changes in the outcome variables; (ii) possible non-linearity of the relationship between amount of time reallocated and estimated changes in the outcome variable, and (iii) possible differences in the relationships at various baseline daily activity levels. These differences stem from the fundamental limitation of traditional isotemporal substitution; it is a statistical technique for real space, requiring the use of standard operations and metrics to be applied in an Euclidean geometry. Daily activity behaviour data, being by nature compositional, do not exist in real space; rather they occupy a simplex sample space. The expression of daily activity behaviour data as *ilr* coordinates overcomes this statistical issue, as the corresponding coordinates occupy real space and follow a Euclidean geometry. Compositional data analysis provides a robust way of including all daily activity behaviours in multivariate statistical models, and thus can provide integrated insight into the associations with adiposity (and likely other health outcomes) of daily activity behaviours. Furthermore, the approach is not limited to pair-wise reallocations between behaviours; types of reallocation can be tailored to match the research question, for example, the reallocation of a fixed duration of time to MVPA at the expense of the remaining daily behaviours in a pro-rata fashion (*one-for-many* reallocation) can be used to explore the health associations of MVPA in relation to the remaining behaviours [12].

Compositional approaches are relatively new in analysing use-of-time data in health research. Future research should use compositional data analysis to explore reallocation relationships with other health outcomes, such as fitness, quality of life, cardiometabolic biomarkers and other disease risk indicators. It is particularly important to explore longitudinal changes in activity compositions associated with maturation, secular changes and interventions, and their relationships with indicators of health. The analyses conducted in the present study represent a basic approach to assessing the relationship between health and compositional isotemporal substitution. Once compositions have been expressed as *ilr* co-ordinates, more complex and potentially useful multivariate methods can be applied, for example, the use of splines or other non-parametric regression models. Furthermore, there is future scope to apply the compositional isotemporal substitution paradigm to other areas such as body composition and daily macronutrient intake.

## Additional files

Additional file 1: Compositional Data Analysis Description and Sample R Code (PDF 2113 kb)

Additional file 2: Characteristics of Included and Excluded Participants (PDF 91 kb)

Additional file 3: Mean Daily Behaviour Composition Across zBMI Categories (PDF 93 kb)

Additional file 4: Compositional Isotemporal Substitution at Weight Status Subgroups (PDF 100 kb)

## Abbreviations

BMI: Body Mass Index
DXA: Dual-energy X-ray absorptiometry
ISCOLE: International Study of Childhood Obesity, Lifestyle and the Environment
LPA: Light Physical Activity
MVPA: Moderate-to-Vigorous Physical Activity
