# Supplementary material for: Adiposity and the isotemporal substitution of physical activity, sedentary time and sleep among school-aged children: a compositional data analysis approach
Source: BMC Public Health. 2018 Mar 2;18:311. doi: 10.1186/s12889-018-5207-1 (PMC5834855; doi:10.1186/s12889-018-5207-1)
Supplement: Supplementary file 1 — Compositional Data Analysis Description and Sample R Code (PDF 2113 kb) [file 12889_2018_5207_MOESM1_ESM.pdf]

Supplementary file 1: Estimation of Differences in Body Fat Percentage associated with Compositional Isotemporal Substitution of Activity Behaviours.

The ilr regression model

As presented in the main document, body fat percentage (%BF) can be estimated for specific daily activity behaviour compositions using the isometric log-ratio (*ilr)* linear multiple regression model. We can estimate %BF (Y_i), for subjects $i=1,2,\ldots n,$ from the *ilr* linear model as follows:

$$Y_i = \beta_0 + \boldsymbol{\beta}^T ilr(\boldsymbol{x}_i) + \varepsilon_i \qquad (1)$$

where $\beta_0$is the intercept, $\boldsymbol{\beta}^T = (\beta_1, \beta_2, \ldots, \beta_{D-1})$ is a vector of coefficients and $\boldsymbol{x}_i = (x_{i1}, x_{i2}, \ldots, x_{iD})^T$ are the D observed composition values (parts of the composition) for subject *i.* The *ilr* function to express the composition as log ratio coordinates is $ilr(.)$ and ε_i is a zero-centred, independently normally distributed random variable with variance σ^2.

Estimating a baseline body fat percentage

Body fat percentage for a baseline composition can be calculated using the estimated parameters $\hat{\beta}_0$ and $\hat{\boldsymbol{\beta}}$ from Equation 1. For example, the mean daily activity behaviour composition can be used as a baseline/reference composition, i.e., $(\overline{\text{Sleep}}, \overline{\text{SED}}, \overline{\text{LPA}}, \overline{\text{MVPA}})$ in minutes, (where $\bar{x}$ represents the geometric mean of x, and SED = sedentary time, LPA = light physical activity, MVPA = moderate-to-vigorous physical activity) can be used to estimate a baseline/reference value for %BF, as shown in Equation 2:

$$\hat{\bar{y}} = \hat{\beta}_0 + \hat{\boldsymbol{\beta}}^T ilr(\overline{\text{Sleep}}, \overline{\text{SED}}, \overline{\text{LPA}}, \overline{\text{MVPA}}) \qquad (2)$$

Estimating body fat percentage for a new composition

We now calculate estimated %BF for a new composition where a fixed duration of time has been reallocated from one behaviour to another, keeping the remaining behaviours constant. For example, when 30 are reallocated from sedentary behaviour to sleep, the new predictive composition is: $\left(\overline{\text{Sleep}} + 30, \overline{\text{SED}} - 30, \overline{\text{LPA}}, \overline{\text{MVPA}}\right)$, which can be used to estimate %BF as follows:

$$\hat{\bar{y}}_{(+30,-30,0,0)} = \hat{\beta}_0 + \hat{\boldsymbol{\beta}}^T ilr\left(\left(\overline{\text{Sleep}} + 30, \overline{\text{SED}} - 30, \overline{\text{LPA}}, \overline{\text{MVPA}}\right)\right) \qquad (3)$$

Estimating the expected difference in body fat percentage between a reallocated composition and the mean composition

The difference between estimates of %BF obtained from Equation 3 and Equation 2 can be determined by subtraction, as below:

$$\hat{\bar{y}}_{(+30,-30,0,0)} - \hat{\bar{y}} \qquad (4)$$

The above approach can be used to estimate change in %BF for any reallocation between compositional parts, using any designated composition of interest as the baseline/reference.

The following R code can be used to calculate the difference in %BF estimated for two compositions.

```
# load the required R package, Compositions
library(Compositions)

# make the composition by binding the components together
comp <- cbind(sleep,SED,LPA,MVPA) #variables are in min/day

# tell R that comp is a compositional variable
comp <- acomp(comp)
```

```r
# make the ilr multiple linear regression model. BF represents
%body fat. ilr() is the default isometric log ratio
transformation included in the Compositions package.
lm <- lm(BF ~ ilr(comp) + ses) #ses, an example covariate

# determine the mean composition
comp.mean <- mean(comp)
# because comp has been designated as a compositional
variable, R calls on mean.acomp() to calculate the
compositional mean (i.e., geometric mean of each component,
then adjusted so all components sum to 1). The mean is
therefore expressed in proportions.

# predict %BF for the mean composition from above, keeping ses
constant at its mean.

mean.pred <- predict(lm, newdata=list(comp=comp.mean,
ses=mean(ses)))

# next, construct a new composition, where 30 min of sedentary
time have been reallocated to sleep. However, here 30 min must
be expressed as a proportion (remember, from above, that
mean.acomp() adjusts the mean to proportions).

# The reallocated time is therefore 30/1440.
new.comp <- acomp(comp.mean  +c(30/1440, -30/1440, 0, 0))

# Now, we predict %BF for the new composition (new.comp),
keeping ses constant at its mean.
pred <- predict(lm, newdata=list(comp=new.comp,
ses=mean(ses)))

# Finally, the estimated difference in %BF for the above time
reallocation is (Equation 4):
pred - mean.pred
```
