# Supplementary material for: Adiposity and the isotemporal substitution of physical activity, sedentary time and sleep among school-aged children: a compositional data analysis approach
Source: BMC Public Health. 2018 Mar 2;18:311. doi: 10.1186/s12889-018-5207-1 (PMC5834855; doi:10.1186/s12889-018-5207-1)
Supplement: Supplementary file 2 — Characteristics of Included and Excluded Participants (PDF 91 kb) [file 12889_2018_5207_MOESM2_ESM.pdf]

Supplementary file 2: Characteristics of Included and Excluded Participants

Table 1: Included and excluded participant characteristics

**Characteristic**		**Included (*n* = 1728)**	**Excluded (*n* = 428)**		***p****
Sex	Male	760 (44%)	212 (50%)		**0.018**
	Female	968 (56%)	216 (50%)		
Number of siblings	0	187 (11%)	31 (7%)		**<0.001**
	1	795 (46%)	124 (29%)		
	2	447(26%)	97 (23%)		
	3	183 (11%)	45 (11%)		
	≥4	116 (7%)	131 (31%)		
Number of parents	0-1	299 (17%)	78 (18%)		0.653
	≥2	1429 (83%)	350 (82%)		
Highest parental education level	Level 1[$]	81 (5%)	17 (5%)	$n^{\#}$ = 316	**0.043**
	Level 2	751 (43%)	167 (53%)		
	Level 3	896 (52%)	132 (42%)		
Activity behaviours; Compositional mean (min/d)	Sleep	558	552	$n^{\#}$ = 109	<0.001
	SED	516	539		
	LPA	304	294		
	MVPA	62	55		
Body fat%; Mean (SD)		20.45 (7.11)	20.88 (7.63)	$n^{\#}$ = 394	0.270

Abbreviations: SED, sedentary time; LPA, light physical activity; MVPA, moderate-to-vigorous physical activity. To test for difference between groups: chi squared was used for categorical data and t-test for continuous data. MANOVA was used for isometric log ratio-transformed compositional time use data. *Bold *P* values indicate statistical significant difference at alpha < 0.05 [$]Parent education levels are: 1 = less than high school or some high school; 2 = completed high school or some post-secondary (e.g. vocational diploma or certificate); 3 = bachelor degree or post-graduate. [#]number of excluded participants with complete data for relevant covariate
