# Supplementary material for: Adiposity and the isotemporal substitution of physical activity, sedentary time and sleep among school-aged children: a compositional data analysis approach
Source: BMC Public Health. 2018 Mar 2;18:311. doi: 10.1186/s12889-018-5207-1 (PMC5834855; doi:10.1186/s12889-018-5207-1)
Supplement: Supplementary file 3 — Mean Daily Behaviour Composition Across zBMI Categories (PDF 93 kb) [file 12889_2018_5207_MOESM3_ESM.pdf]

Supplementary File 3

Table 1: Mean Daily Behaviour Composition Across zBMI Categories.

BOYS					
	n	**Sleep (min/day)**	**SED (min/day)**	**LPA (min/day)**	**MVPA (min/day)**
**Overall**	760	550	510	308	72
**Normal weight[a]**	520	551	506	307	75
**Overweight**	158	549	515	307	70
**Obese**	79	541	526	313	61

GIRLS					
	n	**Sleep (min/day)**	**SED (min/day)**	**LPA (min/day)**	**MVPA (min/day)**
**Overall**	968	564	520	301	55
**Normal[a] weight**	672	567	515	301	57
**Overweight**	207	562	526	298	54
**Obese**	78	543	545	307	45

Abbreviations: SED, sedentary time; LPA, light physical activity; MVPA, moderate-to-vigorous physical activity. Mean values correspond to geometric adjusted to collectively sum 1440 minutes, however due to rounding daily averages presented do not always add to 1440. Weight-status subgroups derived from World Health Organization classification of body mass index z-scores. [a]Mean composition for severe thinness/thinness (WHO z-score ≤2) (*n*= 14) subgroups not presented.

N.B.: No standard deviations are provided, as they are inadequate to describe the multi-variate dispersion of compositional data. Refer to variation matrices below.

Table 2: Variation Matrices of Daily Activity Behaviour Components

	**BOYS: All (*n*=760)**				**GIRLS: All (*n* =968)**			
	**Sleep**	**SED**	**LPA**	**MVPA**	**Sleep**	**SED**	**LPA**	**MVPA**
Sleep	0.00	0.04	0.04	0.13	0.00	0.03	0.04	0.11
SED	0.04	0.00	0.07	0.18	0.03	0.00	0.06	0.15
LPA	0.04	0.07	0.00	0.09	0.04	0.06	0.00	0.10
MVPA	0.13	0.18	0.09	0.00	0.11	0.15	0.10	0.00

	**BOYS: Normal Weight (*n* =520)**				**GIRLS: Normal Weight (*n* =672)**			
	**Sleep**	**SED**	**LPA**	**MVPA**	**Sleep**	**SED**	**LPA**	**MVPA**
Sleep	0.00	0.03	0.04	0.12	0.00	0.03	0.03	0.12
SED	0.03	0.00	0.07	0.16	0.03	0.00	0.06	0.16
LPA	0.04	0.07	0.00	0.08	0.03	0.06	0.00	0.09
MVPA	0.12	0.16	0.08	0.00	0.12	0.16	0.09	0.00

	**BOYS: Overweight (*n* =158)**				**GIRLS: Overweight (*n* =207)**			
	**Sleep**	**SED**	**LPA**	**MVPA**	**Sleep**	**SED**	**LPA**	**MVPA**
Sleep	0.00	0.04	0.03	0.16	0.00	0.04	0.05	0.13
SED	0.04	0.00	0.07	0.19	0.04	0.00	0.07	0.12
LPA	0.03	0.07	0.00	0.10	0.05	0.07	0.00	0.10
MVPA	0.16	0.19	0.10	0.00	0.13	0.12	0.10	0.00

	**BOYS: Obese (*n* =79)**				**GIRLS: Obese (*n* =78)**			
	**Sleep**	**SED**	**LPA**	**MVPA**	**Sleep**	**SED**	**LPA**	**MVPA**
Sleep	0.00	0.03	0.04	0.11	0.00	0.05	0.04	0.11
SED	0.03	0.00	0.05	0.20	0.05	0.00	0.06	0.15
LPA	0.04	0.05	0.00	0.12	0.04	0.06	0.00	0.09
MVPA	0.11	0.20	0.12	0.00	0.11	0.15	0.09	0.00

SED, sedentary time; LPA, light physical activity; MVPA, moderate-to-vigorous physical activity. Weight-status subgroups derived from World Health Organization classification of body mass index z-scores. “Severe thinness” and “thinness” weight status categories (*n*=14) not presented.
