# Supplementary material for: Adiposity and the isotemporal substitution of physical activity, sedentary time and sleep among school-aged children: a compositional data analysis approach
Source: BMC Public Health. 2018 Mar 2;18:311. doi: 10.1186/s12889-018-5207-1 (PMC5834855; doi:10.1186/s12889-018-5207-1)
Supplement: Supplementary file 4 — Compositional Isotemporal Substitution at Weight Status Subgroups (PDF 100 kb) [file 12889_2018_5207_MOESM4_ESM.pdf]

**Supplementary File 4: Compositional Isotemporal Substitution at Weight Status Subgroups**

Table 1. Boys: Estimated difference in body fat% associated with the pair-wise reallocation of 30 minutes between behaviours.

**Normal Weight *n* = 520**

| | Sleep | | SED | | LPA | | MVPA | |
|---|---|---|---|---|---|---|---|---|
| | **△′** | **95% CI** | **△′** | **95% CI** | **△′** | **95% CI** | **△′** | **95% CI** |
| **Sleep** | | | -0.21 | -0.46, 0.05 | -0.53 | -0.87, -0.18 | 2.00 | 1.30, 2.71 |
| **SED** | 0.21 | -0.05, 0.46 | | | -0.33 | -0.64, -0.02 | 2.20 | 1.55, 2.85 |
| **LPA** | 0.49 | 0.16, 0.83 | 0.28 | -0.01, 0.57 | | | 2.49 | 1.70, 3.28 |
| **MVPA** | -1.28 | -1.77, -0.79 | -1.49 | -1.93, -1.06 | -1.81 | -2.40, -1.22 | | |

**Overweight *n* = 158**

| | Sleep | | SED | | LPA | | MVPA | |
|---|---|---|---|---|---|---|---|---|
| | **△′** | **95% CI** | **△′** | **95% CI** | **△′** | **95% CI** | **△′** | **95% CI** |
| **Sleep** | | | -0.20 | -0.46, 0.05 | -0.53 | -0.88, -0.18 | 2.22 | 1.45, 2.99 |
| **SED** | 0.20 | -0.05, 0.46 | | | -0.33 | -0.64, -0.02 | 2.42 | 1.70, 3.14 |
| **LPA** | 0.49 | 0.16, 0.83 | 0.28 | -0.01, 0.57 | | | 2.71 | 1.85, 3.56 |
| **MVPA** | -1.37 | -1.89, -0.85 | -1.58 | -2.04, -1.12 | -1.90 | -2.52, -1.29 | | |

**Obese *n* = 79**

| | Sleep | | SED | | LPA | | MVPA | |
|---|---|---|---|---|---|---|---|---|
| | **△′** | **95% CI** | **△′** | **95% CI** | **△′** | **95% CI** | **△′** | **95% CI** |
| **Sleep** | | | -0.20 | -0.46, 0.05 | -0.52 | -0.87, -0.18 | 2.71 | 1.78, 3.64 |
| **SED** | 0.20 | -0.05, 0.46 | | | -0.32 | -0.62, -0.02 | 2.91 | 2.03, 3.78 |
| **LPA** | 0.49 | 0.16, 0.82 | 0.28 | -0.01, 0.56 | | | 3.19 | 2.19, 4.19 |
| **MVPA** | -1.55 | -2.13, -0.98 | -1.76 | -2.28, -1.24 | -2.08 | -2.74, -1.41 | | |

Abbreviations: **△′,** estimated change in body fat%; CI, confidence interval; SED, sedentary time; LPA, light physical activity; MVPA, moderate-to-vigorous physical activity. Severe thinness and thinness weight status categories (*n*=3) not presented. Note: Difference in body fat% is estimated for the reallocation of time from the behaviour in the column to the behaviour in the row, i.e., the first value of -0.21 in Row 1 is the estimated difference in body fat% for the reallocation of 30 minutes from sedentary time to sleep. Analysis adjusted for parental education level and number of parents and siblings.

Table 2. Girls: Estimated difference in body fat% associated with the pair-wise reallocation of 30 minutes between behaviours.

**Normal Weight *n* = 672**

| | Sleep | | SED | | LPA | | MVPA | |
|---|---|---|---|---|---|---|---|---|
| | △′ | 95% CI | △′ | 95% CI | △′ | 95% CI | △′ | 95% CI |
| **Sleep** | | | -0.52 | -0.80, -0.24 | -0.49 | -0.85, -0.12 | 2.33 | 1.27, 3.38 |
| **SED** | 0.52 | 0.24, 0.80 | | | 0.02 | -0.30, 0.34 | 2.83 | 1.82, 3.84 |
| **LPA** | 0.47 | 0.13, 0.82 | -0.06 | -0.37, 0.24 | | | 2.79 | 1.63, 3.94 |
| **MVPA** | -1.21 | -1.83, -0.59 | -1.74 | -2.32, -1.17 | -1.71 | -2.45, -0.97 | | |

**Overweight *n* = 207**

| | Sleep | | SED | | LPA | | MVPA | |
|---|---|---|---|---|---|---|---|---|
| | △′ | 95% CI | △′ | 95% CI | △′ | 95% CI | △′ | 95% CI |
| **Sleep** | | | -0.52 | -0.80, -0.24 | -0.49 | -0.86, -0.12 | 2.54 | 1.40, 3.67 |
| **SED** | 0.51 | 0.23, 0.79 | | | 0.01 | -0.31, 0.33 | 3.04 | 1.94, 4.13 |
| **LPA** | 0.48 | 0.13, 0.83 | -0.05 | -0.36, 0.25 | | | 3.00 | 1.76, 4.24 |
| **MVPA** | -1.27 | -1.92, -0.62 | -1.80 | -2.40, -1.20 | -1.77 | -2.54, -1.01 | | |

**Obese *n* =78**

| | Sleep | | SED | | LPA | | MVPA | |
|---|---|---|---|---|---|---|---|---|
| | △′ | 95% CI | △′ | 95% CI | △′ | 95% CI | △′ | 95% CI |
| **Sleep** | | | -0.51 | -0.79, -0.24 | -0.50 | -0.85, -0.13 | 3.48 | 1.96, 5.00 |
| **SED** | 0.51 | 0.23, 0.79 | | | 0.01 | -0.30, 0.32 | 3.98 | 2.51, 5.45 |
| **LPA** | 0.48 | 0.13, 0.83 | -0.05 | -0.34, 0.25 | | | 3.95 | 2.33, 5.56 |
| **MVPA** | -1.49 | -2.23, -0.75 | -2.02 | -2.71, -1.33 | -1.20 | -2.85, -1.14 | | |

Abbreviations: △′, estimated change in body fat%; CI, confidence interval; SED, sedentary time; LPA, light physical activity; MVPA, moderate-to-vigorous physical activity. Severe thinness and thinness weight status categories (*n*=11) not presented. Note: Difference in body fat% is estimated for the reallocation of time from the behaviour in the column to the behaviour in the row, i.e., the first value of -0.52 in Row 1 is the estimated difference in body fat% for the reallocation of 30 minutes from sedentary time to sleep.
Analysis adjusted for parental education level and number of parents and siblings.
